# Circulating miR-30a-5p as a prognostic biomarker of left ventricular dysfunction after acute myocardial infarction

**DOI:** 10.1038/s41598-018-28118-1

**Published:** 2018-06-29

**Authors:** Agata Maciejak, Edyta Kostarska-Srokosz, Wlodzimierz Gierlak, Miroslaw Dluzniewski, Marek Kuch, Michal Marchel, Grzegorz Opolski, Marek Kiliszek, Krzysztof Matlak, Slawomir Dobrzycki, Anna Lukasik, Agnieszka Segiet, Grazyna Sygitowicz, Dariusz Sitkiewicz, Monika Gora, Beata Burzynska

**Affiliations:** 1 Institute of Biochemistry and Biophysics, Polish Academy of Sciences, Warsaw, Poland; 2 Chair and Department of Cardiology, Hypertension and Internal Medicine, Second Faculty of Medicine, Medical University of Warsaw, Mazovian Bródnowski Hospital, Warsaw, Poland; 3 Cardiology Department, Midtown Medical Center, Mazovia Brodno Hospital, Warsaw, Poland; 4 1st Chair and Department of Cardiology, Medical University of Warsaw, Warsaw, Poland; 5 Department of Cardiology and Internal Diseases, Military Institute of Medicine, Warsaw, Poland; 6 Department of Cardiac Surgery, Medical University of Bialystok, Bialystok, Poland; 7 Department of Invasive Cardiology, Medical University of Bialystok, Bialystok, Poland; 8 1st Faculty of Medicine, Medical University of Warsaw, Warsaw, Poland; 9 Department of Clinical Chemistry and Laboratory Diagnostics, Medical University of Warsaw, Warsaw, Poland

## Abstract

Left ventricular (LV) dysfunction after acute myocardial infarction (AMI) is associated with an increased risk of heart failure (HF) development. Diverse microRNAs (miRNAs) have been shown to appear in the bloodstream following various cardiovascular events. The aim of this study was to identify prognostic miRNAs associated with LV dysfunction following AMI. Patients were divided into subgroups comprising patients who developed or not LV dysfunction within **six** months of the infarction. miRNA profiles were determined in plasma and serum samples of the patients on the first day of AMI. Levels of 14 plasma miRNAs and 16 serum miRNAs were significantly different in samples from AMI patients who later developed LV dysfunction compared to those who did not. Two miRNAs were up-regulated in both types of material. Validation in an independent group of patients, using droplet digital PCR (ddPCR) confirmed that miR-30a-5p was significantly elevated on admission in those patients who developed LV dysfunction and HF symptoms six months after AMI. A bioinformatics analysis indicated that miR-30a-5p may regulate genes involved in cardiovascular pathogenesis. This study demonstrates, for the first time, a prognostic value of circulating miR-30a-5p and its association with LV dysfunction and symptoms of HF after AMI.

## Introduction

The development of heart failure (HF) after acute myocardial infarction (AMI) is a consequence of left ventricular (LV) remodeling, a process which causes molecular, cellular, and interstitial changes that are revealed clinically as aberrations in the size, shape, and functioning of the heart^1–3^. The mechanisms of LV dysfunction are complex, the most important changes including the β-adrenergic pathway, calcium transport, contractile proteins, collagen accumulation, cell death, increased oxidative stress, energy deficit, membrane and matrix proteins, and ventricular geometry. In addition, LV remodeling is associated with a higher prevalence of cardiac rupture, arrhythmia, and aneurysm formation after infarction^4^. Despite the recent progress in invasive and pharmacological treatment, LV remodeling is observed in approximately 30% of patients with AMI^5^. Early prediction of LV remodeling, which would allow the selection of patients at a high risk of developing post-infarcted HF, is therefore important therapeutically and any improvement in its accuracy and/or simplicity by the identification of novel biomarkers would be highly advantageous.

Recent studies have revealed important roles for microRNAs (miRNAs) in the pathogenic mechanisms leading to HF, such as cardiac remodeling, hypertrophy, apoptosis, and hypoxia^6–8^. miRNAs are a class of small (~20–25 nucleotides), non-coding RNA molecules which regulate gene expression by promoting mRNA degradation or by inhibiting translation^9,10^. Since miRNAs can be detected in the circulation, they are attractive as potential biomarkers for the diagnosis and prognosis of diverse diseases. In contrast to cancer research, only few studies focused on the use of miRNAs in prognosis and/or diagnosis of acute heart failure have been published^11^.

The aim of the present study was to compare the profiles of circulating miRNAs in serum and plasma samples collected on admission from AMI patients: those who developed LV dysfunction and symptoms of HF during the first six months after AMI and those who did not. Using the droplet digital PCR (ddPCR) method, we validated the level of miR-30a-5p in serum samples from an independent group of AMI patients. This analysis indicated that an elevated level of miR-30a-5p is a good predictor of the future development of LV dysfunction and HF symptoms, following AMI.

## Results

### Patient characteristics

In the present study, a total of 99 patients presenting with STEMI (ST Elevation Myocardial Infarction) were assigned to two groups: the study group (n = 14) and the validation group (n = 85). The mean age of the patients was 52.5 years (range 45.0‒62.8) and 59.0 years (range 50.0–65.0), respectively. Clinical characteristics of the study and validation groups are shown in Table 1. Apart from BMI and hypertension (p < 0.05), there were no significant differences between the two groups.

**Table 1:** Clinical characteristics of patient groups.

Characteristic	Study group (n = 14)	Validation group (n = 85)	p-value
Gender (female/male)	2/12 (14.3%/85.7%)	11/74 (12.9%/87.1%)	>0.999
Age (years)	52.5 [45.0‒62.8]	59.0 [50.0‒65.0]	0.226
BMI (kg/m^2^)	25.6 [25.2‒27.5]	28.7 [26.2‒31.7]	0.026
Smoking	6 (42.9%)	51 (60.7%)	0.336
Diabetes	2 (14.3%)	14 (16.7%)	>0.999
Hypertension	3 (21.4%)	55 (64.7%)	0.006
Hypercholesterolemia	7 (50%)	40 (47.6%)	>0.999
Previous MI	0 (0.0%)	3 (3.6%)	>0.999
Anterior MI	7 (53.8%)	23 (27.1%)	0.102
NT-proBNP (pg/ml) admission	1006.3 [431.0‒1820.2]	860.0 [412.8‒1545.8]	0.587
NT-proBNP (pg/ml) follow-up	193.8 [69.5‒786.0]	174.5 [68.0‒318.5]	0.427
LVEF (%) admission	47.0 [42.2‒56.5]	50.0 [44.2‒55.0]	0.715
LVEF (%) follow-up	57.0 [42.0‒66.0]	56.0 [49.0‒60.0]	0.633
Medication			
Aspirin	14 (100%)	84 (100.0%)^*^	NA
Clopidogrel	13 (92.9%)	74 (88.1%)	>0.999
Beta blockers	14 (100%)	76 (90.5%)	0.597
ACE inhibitors	14 (100%)	68 (81.0%)	0.117
Statins	14 (100%)	82 (97.6%)	>0.999
Diuretics	6 (42.9%)	23 (27.4%)	0.342

Data are presented as number (%) or median [first quartile - third quartile]. P < 0.05 was considered to indicate a statistically significant difference. ^*^Data were only available for n = 84 patients; these numbers were used to calculate percentages. BMI – Body Mass Index; LVEF – Left Ventricular Ejection Fraction; MI – myocardial infarction; NT-proBNP – N-terminal pro-B-type natriuretic peptide.

The study group was divided into subgroups comprising patients who developed LV dysfunction and HF symptoms during the six months following AMI (LVEF ≤ 50%, NT-proBNP ≥ 150 pg/ml; termed the HF group, n = 7) and those who did not (LVEF ≥ 60%, NT-proBNP ≤ 100 pg/ml; termed the non-HF group, n = 7). The same criteria were used to differentiate patients from the validation group (HF group, n = 22, and non-HF group, n = 12). Patients who did not meet the above criteria for either LVEF or NT-proBNP or both were assigned to a “middle” validation group (termed the mod-HF group, n = 51). Patients with stable coronary artery disease (CAD) served as a control group.

### Differentially expressed miRNAs in the serum and plasma of patients with LV dysfunction after AMI

The first miRNA profiling was performed in the study group, on plasma samples collected from AMI patients upon admission. The obtained results were then compared between patients who developed LV dysfunction and symptoms of HF during six months of follow-up (n = 7) and patients from the non-HF group (n = 7). Following normalization of raw data, a total of 14 miRNAs (6 up- and 8 down-regulated) were found to be significantly differentially expressed in the HF group versus the non-HF group (p < 0.05). The second miRNA profiling was performed on serum samples derived from randomly selected patients from the validation group (n = 5 for the HF group and n = 5 for non-HF). Here, 16 miRNAs (13 up- and 3 down-regulated) showed significantly altered levels in HF patients compared with non-HF. Two differentially expressed miRNAs, miR-30a-5p and miR-99b-5p, were common to both comparisons (Table 2) and showed similar differences in both types of biological material: miR-30a-5p − 4.840, p = 0.0444 in plasma and 5.169, p = 0.0430 in serum; miR-99b-5p − 2.802, p = 0.0205 in plasma and 2.717, p = 0.0233 in serum.

**Table 2:** miRNAs showing significantly different levels in plasma or serum in HF versus non-HF AMI patients.

Plasma			Serum		
miRNA	Fold change	p-value	miRNA	Fold change	p-value
miR-18a-3p	6.193	0.0003	miR-195–5p	6.277	0.0311
**miR-30a-5p**	4.840	0.0444	miR-378a-3p	5.594	0.0213
miR-99a-5p	3.525	0.0220	**miR-30a-5p**	5.169	0.0430
**miR-99b-5p**	2.802	0.0205	miR-22-3p	3.415	0.0003
miR-130a-3p	1.664	0.0261	miR-365a-3p	3.331	0.0296
miR-106a-5p	1.222	0.0385	miR-29c-3p	3.299	0.0263
miR-29a-3p	−1.646	0.0451	miR-30d-5p	3.006	0.0140
let-7g-5p	−1.832	0.0111	**miR-99b-5p**	2.717	0.0233
miR-29b-3p	−1.854	0.0239	miR-152	2.266	0.0217
miR-142-3p	−1.991	0.0251	let-7d-3p	2.056	0.0014
miR-150-5p	−2.731	0.0104	miR-30e-5p	1.808	0.0018
miR-338-3p	−3.103	0.0454	miR-222-3p	1.783	0.0050
miR-361-3p	−3.951	0.0193	miR-590-5p	1.412	0.0071
miR-146b-5p	−6.311	0.0250	let-7a-5p	−1.507	0.0311
			miR-103a-3p	−1.602	0.0246
			miR-107	−1.604	0.0174

Data are presented as the fold change in the HF group compared to the non-HF group. P-values were calculated using unpaired two-tailed Student’s t-test. Only miRNA species with p < 0.05 are shown. miRNAs common to both types of biological material are indicated in bold.

### Validation of profiling data using ddPCR and development of miRNA biomarkers for post-myocardial infarction LV dysfunction

Serum samples from patients from the validation group (n = 22 HF, n = 51 mod-HF and n = 12 non-HF) and a CAD control group (n = 12) were subjected to ddPCR, focusing on the two miRNAs common for the earlier serum and plasma comparisons, miR-99b-5p and miR-30a-5p. The results for miR-99b-5p turned out to be unreliable due to problems with droplet cluster separation and consequently this miRNA species was excluded from further consideration. For miR-30a-5p, however, the ddPCR analysis confirmed its significantly higher median level in HF than in non-HF patients (3.34-fold; p = 0.0166). Notably, patients who did not meet the criteria for inclusion into either of these two groups (mod-HF group) showed a significantly lower median level of miR-30a-5p than patients from the HF group (1.93-fold; p = 0.0234). In turn, they presented a higher median level than non-HF patients, but this difference was not statistically significant (1,73-fold; p = 0.0786).

**Figure 1:**
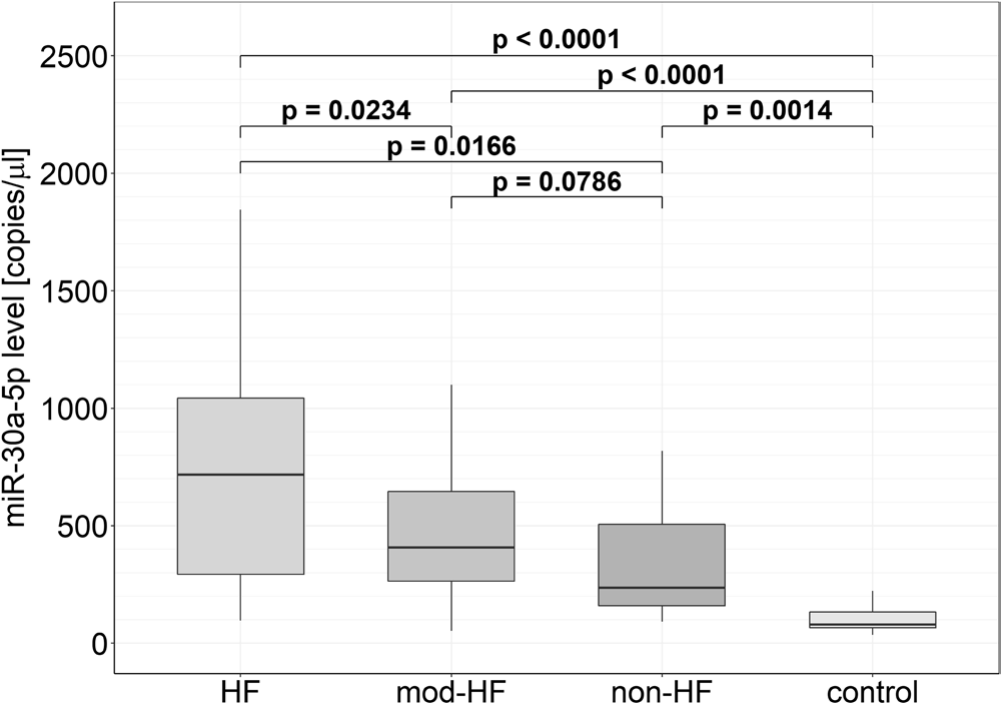
Levels of miR-30a-5p in serum samples from HF, mod-HF, non-HF and CAD control groups. Box plots show the levels of miR-30a-5p measured by ddPCR and expressed in copies per microliter of serum. The bottom and top of each box represents the 1^st^ and 3^rd^ quartiles of the data, respectively. The median (HF: 787.6 [348.8, 1216.0], mod-HF: 407,4 [264.0, 645.3], non-HF: 235.8 [159.9, 505.6], CAD: 79.2 [65.5, 133.2]) is shown as a solid line across the box.

Compared with the CAD control group, miR-30a-5p was significantly more abundant not only in HF patients (9.95-fold; p < 0.0001), but also in mod-HF (5.14-fold; p < 0.0001) and non-HF (2.98-fold; p = 0.0014) patients (Fig. 1).

To evaluate the relationship between the level of miR-30a-5p upon AMI and LV dysfunction development, we calculated Spearman’s rank correlation coefficients for the miR-30a-5p level on admission and NT-proBNP and LVEF on admission and six months after AMI (Supplementary Table S1). The results showed a statistically significant negative monotonic correlation between miR-30a-5p level on admission and the LVEF value six months after AMI.

**Figure 2:**
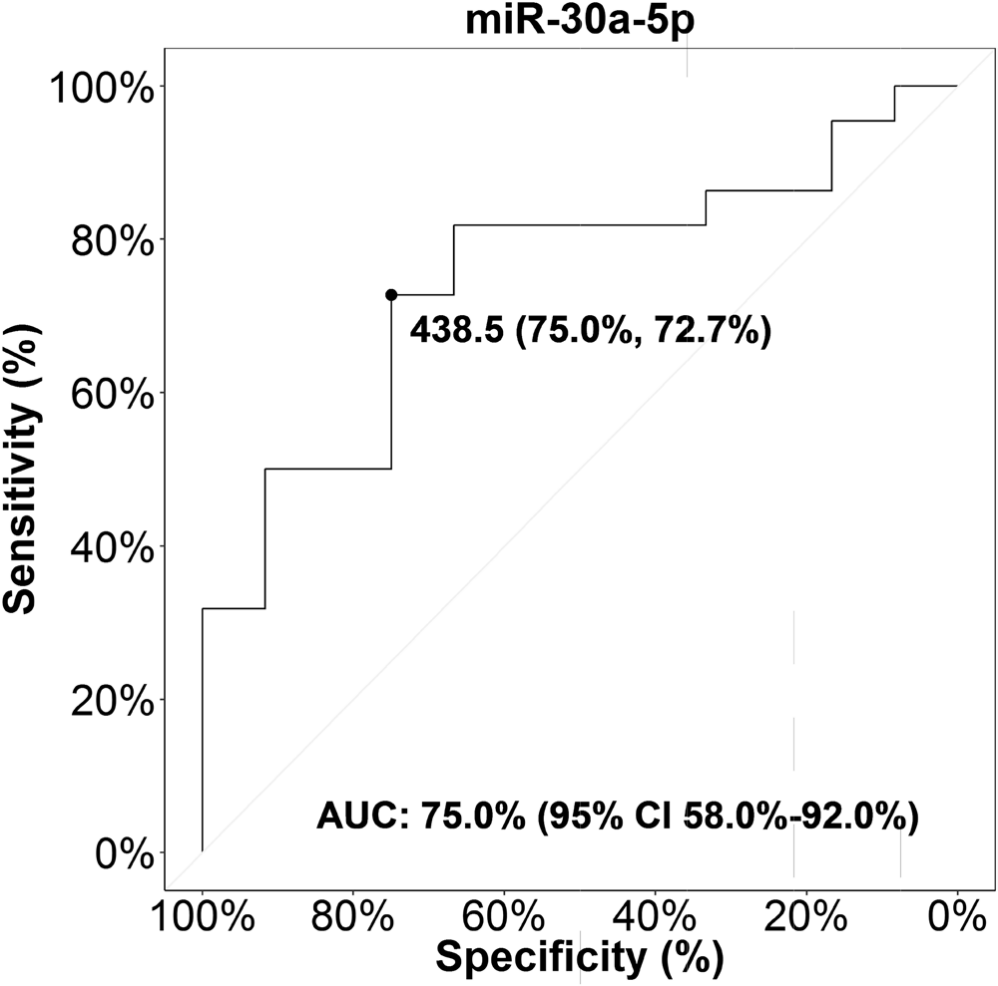
ROC curve for miR-30a-5p. ROC curve analysis of miR-30a-5p level in serum (copies/µl) of n = 22 HF and n = 12 non-HF patients. AUC, area under the curve; CI, confidence interval; ROC, receiver operating characteristic.

To determine the prognostic value of serum miR-30a-5p, ROC and AUC were calculated by using the miRNA copy number/µl serum for the HF and non-HF groups. For the discrimination of HF versus non-HF patients, the AUC for miR-30a-5p was 75% (95% confidence interval: 58–92%). The ROC curves yielded an optimal cut-off value of 438.5 copies/µl, with a sensitivity of 72.7% and a specificity of 75.0% (Fig. 2).

### *In silico* miRNA functional analysis

To predict possible functions of miR-30a-5p, especially in LV remodeling, its potential targets were identified by bioinformatic analysis in two parallel procedures, carried out individually for cardiac tissue and for whole blood. Two sets of human genes were analyzed for potential targets of miR-30a-5p: 8223 genes expressed in heart tissues and 4146 genes expressed in whole blood. Of these, 310 and 149 were identified as potential targets of miR-30a-5p in the heart and whole blood, respectively (Supplementary Table S2). Notably, 138 targets were common to both tissues which remains in accordance with the finding that the heart and blood transcriptomes are largely shared^12^.

**Figure 3:**
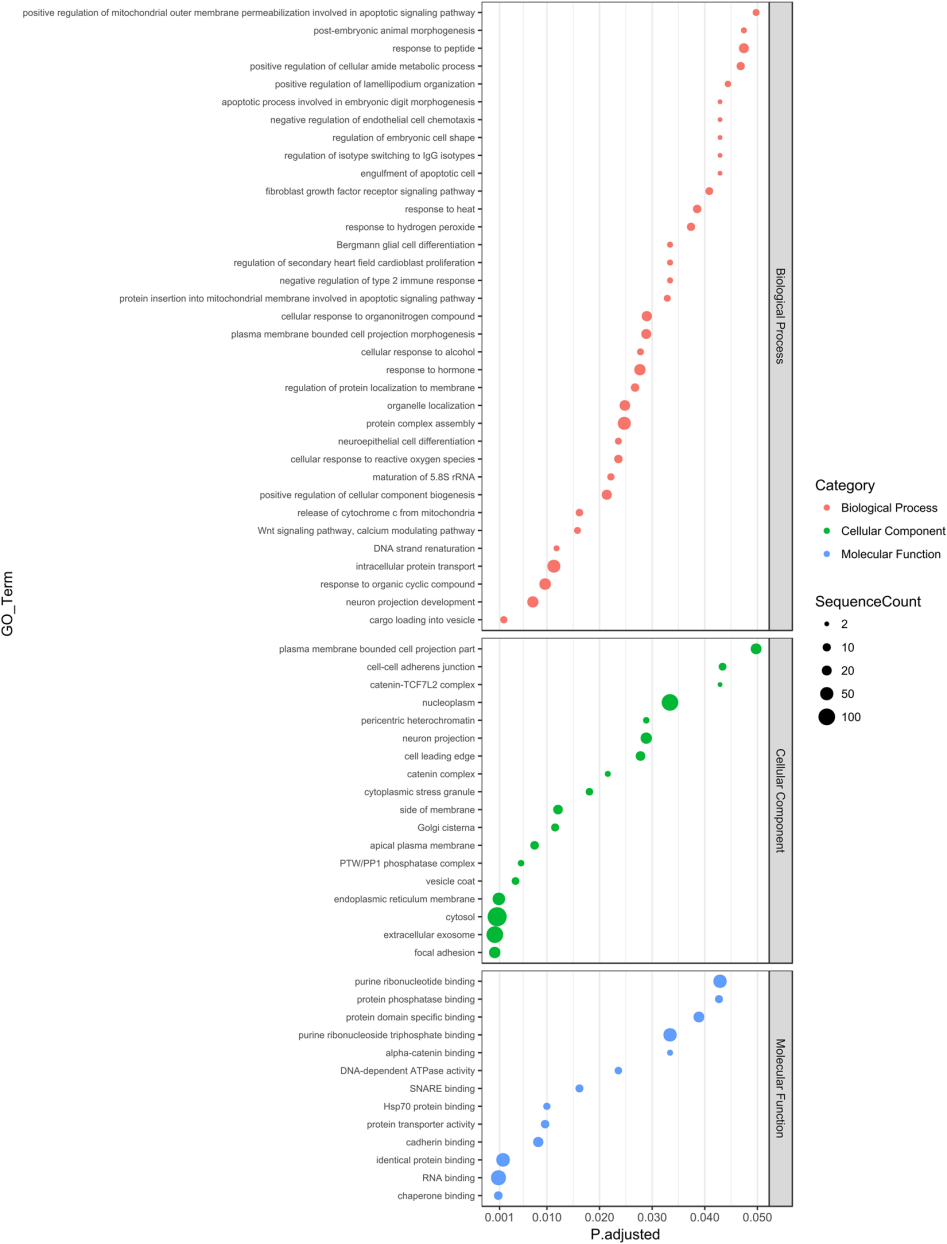
Enriched GO terms for predicted miR-30a-5p targets expressed in the heart. Putative gene targets of miR-30a-5p expressed in the heart were predicted as detailed in Materials and Methods and subjected to a GO enrichment analysis. All genes expressed in the heart served as the background population set. The figure shows enriched GO terms (lowest hierarchy level) from the “Biological Process”, “Molecular Function” and “Cellular Component” categories. The sizes of the dots reflect the number of targets annotated by a given GO term. Only terms with adjusted p-value < 0.05 are shown.

**Figure 4:**
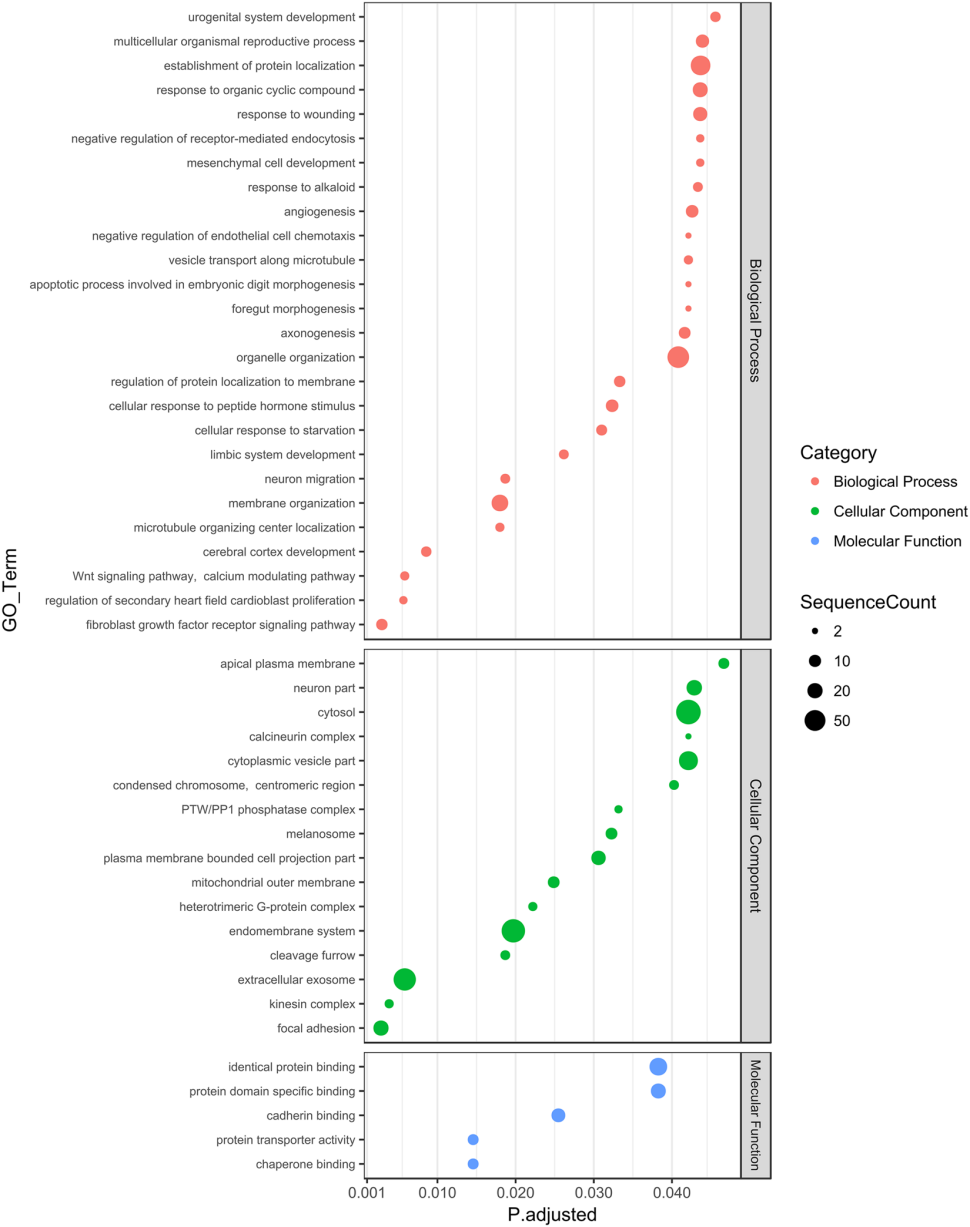
Enriched GO terms for predicted miR-30a-5p targets expressed in whole blood. Putative gene targets of miR-30a-5p expressed in blood were predicted as detailed in Materials and Methods and subjected to a GO enrichment analysis. All genes expressed in the whole blood served as the background population set. The figure shows enriched GO terms (lowest hierarchy level) from the “Biological Process”, “Molecular Function” and “Cellular Component” categories. The sizes of the dots reflect the number of targets annotated by a given GO term. Only terms with adjusted p-value < 0.05 are shown.

The potential miR-30a-5p target genes were characterized functionally by performing GO and KEGG pathway enrichment analysis. For the heart-expressed target genes, 237 GO terms were enriched (Fig. 3 and Supplementary Table S3), and for the potential whole blood target genes 179 GO terms were enriched (Fig. 4 and Supplementary Table S4). Among them were shared terms related to the pathophysiology of the cardiovascular system such as the Wnt signaling pathway (GO:0016055), the calcium modulating pathway (GO:0007223), the fibroblast growth factor signaling pathway (GO:0008543), regulation of secondary heart field cardioblast proliferation (GO:0003266), the PTW/PP1 phosphatase complex (GO:0072357), cadherin binding (GO:0045296), and chaperone binding (GO:0051087). In addition, several terms associated with oxidative stress and apoptotic signaling in mitochondria were enriched for the heart-expressed targets, whereas terms related to blood vessel development and morphogenesis as well as heart formation were enriched for the blood-expressed targets.

**Figure 5:**
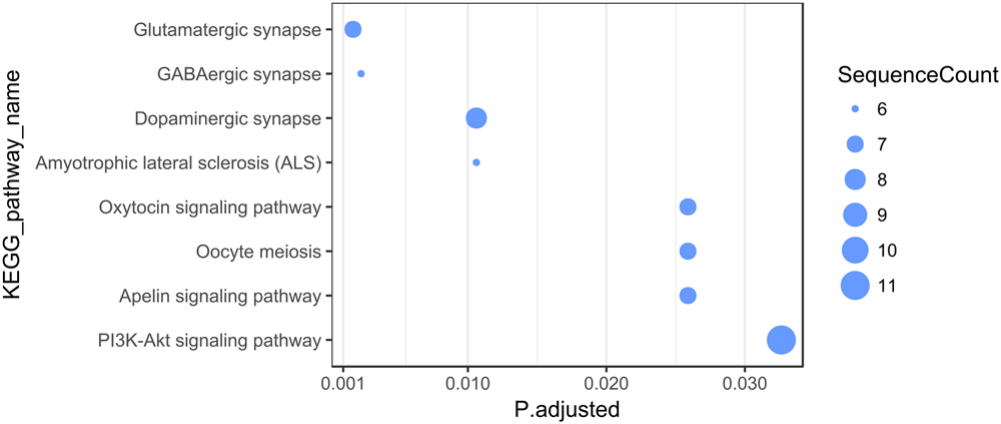
Enriched KEGG pathways for predicted miR-30a-5p targets expressed in whole blood. The KEGG pathway enrichment analysis was performed as detailed in Materials and Methods. Putative gene targets of miR-30a-5p expressed in whole blood were used as the target set. All genes expressed in whole blood served as the background population set. The sizes of the dots reflect the number of targets mapped on a given KEGG pathway. The p-value/Q-value thresholds were set at 0.05.

The KEGG pathway enrichment analysis showed that the heart-expressed target genes for miR-30a-5p may be associated with the “Amyotrophic lateral sclerosis (ALS)” pathway (hsa05014; adjusted p-value 0.005). The target genes expressed in whole blood may, in turn, be involved in eight biological pathways, including the “Apelin signaling pathway” (hsa04371, adjusted p-value 0.025), “Dopaminergic synapse” (hsa04728, adjusted p-value 0.010), “Oxytocin signaling pathway” (hsa04921, adjusted p-value 0.025) and “PI3K-Akt signaling pathway” (hsa04151, adjusted p-value 0.032) with the highest number of mapped genes (Fig. 5). Notably, the “Apelin signaling pathway” and “PI3K-Akt signaling pathway” were also amply represented among the heart-expressed targets, with 8 and 13 genes, respectively, albeit they were not statistically significantly enriched (data not shown).

## Discussion

Despite the recent advances in the therapy and management of cardiovascular diseases, post-myocardial infarction HF remains a serious clinical, epidemiological and economic problem^13^. The molecular mechanisms underlying post-myocardial infarction LV remodeling are not yet fully understood. The cardiovascular biomarkers used in contemporary clinical practice are not accurate prognostic indicators of LV remodeling and HF after acute MI^14,15^. Thus, the identification of new, more reliable, prognostic biomarkers allowing early confident identification of patients who display a high risk of developing post-infarction HF would be very desirable. miRNAs perform essential functions in the pathogenesis of cardiovascular diseases. Their presence and stability in biological fluids has led to extensive investigation of their possible use as potential non-invasive biomarkers^16^. miRNA profiling which can be done in easily available serum or plasma samples allows better understanding of the changes that occur in the post-infarcted myocardium. However, special attention must be paid to the selection of the type of specimen for profiling since substantial quantitative and qualitative differences between serum and plasma have been noted^17,18^. In this study the profile of circulating miRNAs was determined in both serum and plasma samples from patients with deteriorated LV function following AMI and from patients exhibiting no such deterioration in order to select potential prognostic miRNAs. Of the 179 analyzed miRNAs, levels of 14 miRNAs from plasma samples and 16 from serum differed significantly between the two patient groups. Two miRNAs were up-regulated in both types of material. Notably, among the differentially expressed miRNAs were miR-29a-3p and miR-150–5p, shown earlier to be strongly associated with LV dysfunction^19–21^.

By performing miRNA profiling followed by an independent ddPCR evaluation, we established that miR-30a-5p was significantly elevated on admission in patients who during the following six months developed LV dysfunction and HF symptoms, when compared to non-HF, mod-HF, and CAD patients. ROC curve analysis revealed a potential prognostic value for elevated miR-30a-5p levels in the early identification of patients at risk of LV dysfunction. miR-30a-5p has previously been implicated in cancer progression including non-small cell lung cancer^22^, colorectal cancer^23^, urothelial carcinoma^24^ and other types of cancer. In recent years, accumulating evidence has also revealed an involvement of the miR-30 family in cardiovascular pathophysiology^6^. It has been observed that miR-30a-5p expression is increased in the acute phase of myocardial infarction^25^. Other studies showed that the miR-30a level was elevated in the plasma of patients with LV hypertrophy and HF^26,27^. In a rat model of cardiac hypertrophy induced by transverse abdominal aortic constriction, Pan *et al*.^26^ demonstrated that miR-30a influenced autophagy in cardiomyocytes through regulation of beclin-1 expression. Additionally, cystathionine-γ-lyase has been identified as a target of the miR-30 family, indicating that these miRNAs participate in the protection against ischemic injury by regulating hydrogen sulfide production^28^. It has also been found that miR-30a-5p and miR-30c-5p target genes are involved in the regulation of apoptosis in myocardial cells^29^.

In an attempt to provide better understanding of the regulatory functions of miR-30a-5p, we identified key pathways involving genes potentially targeted by this miRNA. The functional analysis was limited to cardiac tissue and whole blood. Although they have diverse functions, over 80% of the peripheral blood transcriptome is shared with the heart transcriptome^12^. Our GO and KEGG enrichment analyses indicated pathways previously found to be of great importance in cardiovascular pathogenesis, especially diverse signal-transduction pathways which relay signals driving cardiac hypertrophy. Notably, there is substantial overlap and cross-talk between these pathways. One of them, the Wnt signaling pathway is known to be involved in cardiac remodeling, angiogenesis and vascular inflammation leading to atherogenesis^30,31^. Interestingly, a link between miR-30 family members and the Wnt pathway has been established in multiple myeloma^32^. Another signal-transduction pathway found in our analyses, the fibroblast growth factor signaling, plays a crucial role in cardiac remodeling via distinct mechanisms including, inter alia, the PI3K/AKT pathway^33,34^. The PI3K/AKT pathway in turn is regulated by the apelin signaling pathway, both identified in our KEGG analysis. It has been shown that up-regulation of miR-30a-5p in lung cancer cells reduces expression of the PI3K regulatory subunit PIK3R2 to induce cell apoptosis and inhibit cell invasion and migration^35^. It is also worth noting that we identified a potential regulatory role of miR-30a-5p in oxytocin signaling. The action of oxytocin in the cardiovascular system is mediated by nitric oxide and atrial natriuretic peptide (ANP), leading to anti-inflammatory and cardioprotective effects^36^. In view of the above *in silico* results, experimental studies should be carried out to confirm the predicted regulation of individual genes and their corresponding pathways by miR-30a-5p.

Reliable quantitation of circulating plasma miRNAs requires the use of very sensitive and accurate methods. Therefore, we verified the miRNA up-regulation in the validation group using a QX200™ Droplet Digital™ PCR System. The ddPCR approach has many advantages over classical RT-qPCR, among others it allows detection of low concentrations of a target without the need for reference miRNAs and standard curves^37^. In accordance, our results demonstrate that ddPCR is a highly sensitive, precise, and reproducible method for quantification and validation of molecular biomarkers for clinical application.

Some limitations of the current study need to be considered. We cannot exclude the possibility that the level of miR-30a-5p could be affected by differences in the time of blood collection. In this regard, a time-course analysis of miR-30a-5p expression after AMI symptom onset could improve diagnostic performance. Further, our study involved only a 6-month follow-up, and a longer prospective study could provide additional information on the clinical outcome. Although the use of multicenter cohorts is a strength of our study, we must acknowledge that the sample size was limited. Thus, our findings can only be considered a foundation for further comprehensive and large-scale assessment of the clinical usefulness of miR-30a-5p as a prognostic biomarker of HF development after myocardial injury.

In conclusion, our study has demonstrated, for the first time, the prognostic value of miR-30a-5p overexpression in plasma and serum as a biomarker of LV dysfunction in AMI patients. The bioinformatics analysis has indicated that miR-30a-5p is likely involved in metabolic pathways relevant to cardiovascular pathogenesis. In addition, we have confirmed the value of the ddPCR method for detecting specific miRNA in serum/plasma samples for diagnostic purposes.

## Materials and Methods

### Patients

Two groups of patients with ST-segment elevation myocardial infarction (STEMI) treated with primary percutaneous coronary intervention (PCI) were enrolled in this study. The study group comprised 14 patients admitted to the First Chair and Department of Cardiology of the Medical University of Warsaw. The validation group comprised 85 patients admitted to the Chair and Department of Cardiology, Hypertension and Internal Medicine of the Medical University of Warsaw, First Chair and Department of Cardiology of the Medical University of Warsaw, Department of Cardiac Surgery and Department of Invasive Cardiology, Medical University of Bialystok. The control group included 12 age- and sex-matched individuals selected from a cohort of patients with stable coronary artery disease (CAD) and no history of acute myocardial infarction (AMI). The whole group of CAD patients had been characterized in our previous study^38^. The clinical outcome of the STEMI patients was determined by the left ventricular ejection fraction (LVEF) and N-terminal pro-B-type natriuretic peptide (NT-proBNP) measured six months after AMI. Patients from the study and validation groups were divided into subgroups comprising patients who developed LV dysfunction and symptoms of HF during the six months following AMI (LVEF ≤ 50%, NT-proBNP ≥ 150 pg/ml; the HF group) and those who did not (LVEF ≥ 60%, NT-proBNP ≤ 100 pg/ml; the non-HF group). Patients who did not meet the criteria for one or both parameters were assigned to a “middle” validation group (the mod-HF group). This study has been approved by the Ethics Committees of the Medical University of Warsaw and the Medical University of Bialystok, and all patients provided written informed consent before participation.

### Blood sample collection, serum/plasma separation

Venous whole blood samples (4-8 ml) were drawn from the patients diagnosed with STEMI at two time points: on the first day of AMI (admission) and six months following AMI, using standard phlebotomy techniques. For the study group, plasma was isolated using BD Vacutainer® CPT™ glass tubes with sodium citrate (BD Biosciences, Franklin Lakes, NJ, USA) following the manufacturer’s protocol. For the validation and CAD control groups, blood samples were drawn into serum separator tubes (Profilab s.c., Warsaw, Poland) according to the manufacturer’s instructions. The plasma and serum samples were stored at −80 °C until analysis. Assessment of hemolysis in the samples was performed as described previously^39^.

### miRNA isolation, reverse transcription

Total RNA including miRNA was isolated from 200 μl of plasma/serum using the miRCURY™ RNA Isolation kit - Biofluids (Exiqon A/S, Vedbaek, Denmark) according to the manufacturer’s protocol. MS2 bacteriophage RNA carrier (Roche Diagnostics GmbH, Mannheim, Germany) and a mix of three synthetic RNA spike-ins (UniSp2, UniSp4 and UniSp5; Exiqon A/S) were added to the samples just before purification at concentrations recommended by the manufacturer. cDNA was synthesized from purified miRNA using the miRCURY™ LNA™ microRNA PCR, Polyadenylation and cDNA synthesis kit II from Exiqon A/S with the addition of two spike-ins (UniSp6 and cel-miR-39–3p; Exiqon A/S) according to the manufacturer’s instructions.

### miRNA RT-qPCR profiling and data analysis

Quantification of miRNA levels in plasma and serum samples was performed using the Serum/Plasma Focus microRNA PCR panel, Version V3 (Exiqon A/S). RT-qPCR reactions were performed using ExiLENT SYBR® Green master mix (Exiqon A/S) according to the manufacturer’s instructions. Negative controls (no template) were performed and profiled in an identical manner as for the samples. The amplification was performed in a LightCycler® 480 Real-Time PCR system (Roche Diagnostics, Basel, Switzerland). The amplification curves were analyzed using the Roche LC software (version 1.5), both for determination of Cp values (by the second derivative method) and for melting curve analysis. GenEx software, version 6.0 (MultiD Analyses AB, Göthenburg, Sweden) was used for data pre-processing including inter-plate calibration, quality control analysis, setting cut-offs for negative control and microRNA Cq values. Only miRNA species with a Cp value < 37 and at least 5 points below the negative control Cp value were included into the data analysis. For the profiling study, the expression data were normalized to a global mean. A logarithmic transformation (log_2_) was used to normalize the expression data at the profiling stage and an unpaired, two-tailed Student’s t-test was performed to determine significance of differences.

### Droplet digital PCR

The quantity of selected miRNAs was verified in serum samples by ddPCR using QX200™ Droplet Digital™ PCR System (Bio-Rad, CA, USA). cDNA synthesized as above was diluted 20-fold, and 9 µl was assayed in a 20-µl reaction volume according to the manufacturer’s protocol for microRNA PCR profiling using microRNA LNA™ PCR primer sets (Exiqon A/S, Denmark) and EvaGreen (Bio-Rad, CA, USA) with the QX200™ Droplet Digital™ PCR System (Bio-Rad, CA, USA) and thermal cycling conditions recommended for EvaGreen assays. After PCR, plates were loaded into a QX200™ Droplet Reader (Bio-Rad, CA, USA) to count PCR-positive and PCR-negative droplets. ddPCR results were analyzed with QuantaSoft™ software v.1.7.4.0917 and QuantaSoft™ Analysis Pro software v.1.0.596 (Bio-Rad, CA, USA). A no template control (NTC) was included in every assay. The miRNA concentrations were calculated using Poisson statistics and background-corrected based on the NTC. Absolute miRNA levels were initially computed in copies/μl PCR reaction and then corrected for the input amount of serum; final results were presented as copies/µl serum.

### miRNA target prediction and functional annotation

To predict possible functions of miR-30a-5p in the heart and blood, its potential targets were identified and annotated. First, genes expressed in heart tissues were collected from the EMBL-EBI Expression Atlas^40^, specifically from five projects: 1) the FANTOM5 project (TPM > 10); 2) the Genotype-Tissue Expression (GTex) project (TPM > 10); 3) the Illumina Body Map (TPM > 10); 4) the ENCODE project (TPM > 10) and 5) the Protein Atlas. Genes expressed in whole blood were also collected from the EMBL-EBI Expression Atlas^40^, specifically from the GTex project (TPM > 10). Then, the 3′UTR sequences of the genes of interest, separately for the heart and blood, were searched for miR-30a-5p targets by the tools4miRs meta-server^41^ using four prediction algorithms: PITA v6^42^, miRanda (Release 2010)^43^, RNA22 v2.0^44^ and miRmap (Release 2013)^45^. The prediction results were supplemented with miRNA target predictions from TargetScan v7.1^46^ and with validated targets from Tarbase v6.0^47^, miRecords v4^48^ and the miRTarBase v7.0^49^ database. Unique miRNA:target pairs expressed in heart tissues/whole blood and predicted by at least three out of the five target prediction algorithms used, or present in at least one of the validated target databases searched, were considered for further analyses. Functional annotation was carried out separately for the miR-30a-5p targets expressed in heart tissues and in whole blood. Gene Ontology (GO) annotation was performed using the Blast2GO software (www.blast2go.com). The GO term enrichment analysis was done with the use of Ontologizer^50^ and an adjusted p-value cutoff set at 0.05; the predicted miR-30a-5p targets were taken as the study set and all genes expressed in heart tissues or whole blood were taken as the population set. The Kyoto Encyclopedia of Genes and Genomes (KEGG) pathway mapping was carried out on-line with the KEGG Mapper service (www.genome.jp/kegg/tool/map_pathway2.html). Human KEGG metabolic pathways served as reference. The KEGG pathway enrichment analysis was performed with the clusterProfiler R package^51^, with study and population sets as GO enrichment analysis. The p-value/Q-value thresholds were set at 0.05.

### Statistical analysis

For categorical variables, the number and percentage of occurrences are reported. Categorical variables were compared using the Fisher test or chi-squared test, depending on the size of the categories. The distribution of continuous variables was first evaluated with the Shapiro-Wilk test and for normally distributed variables the mean and standard deviation (SD) are reported, otherwise the median and the 25th and 75th percentiles (Q1 and Q3) are given. Normally distributed continuous variables were compared with the Student’s t-test, or ANOVA test if more than two variables were compared, otherwise the Mann-Whitney test or Kruskal-Wallis test were used, respectively. Bonferroni correction was used for multiple comparisons. The association between miR-30a-5p level on admission and clinical parameters (NT-proBNP, LVEF) measured on admission and six months after AMI was assessed using Spearman’s rank correlation coefficient and its p-value. The significance level was set at 0.05 and two-sided tests were used. To assess the discriminatory power of the selected miRNA as HF predictor, a receiver operating curve (ROC) was constructed and the area under the curve (AUC) was calculated with a 95% confidence interval. The cut-off value was defined as the number of biomarker copies/µl of serum that corresponds to the point on the ROC curve closest to the (0,1) point. Specificity and sensitivity for the cut-off value are reported. The analysis was performed with the R statistical software, version 3.4.0^52^.

### Data availability

The datasets generated and/or analyzed during the current study are available from the corresponding author on reasonable request.

## Electronic supplementary material

Supplementary information

Table S1

Table S2

Table S3

Table S4
