# Supplementary material for: Circulating miR-30a-5p as a prognostic biomarker of left ventricular dysfunction after acute myocardial infarction
Source: Sci Rep. 2018 Jun 29;8:9883. doi: 10.1038/s41598-018-28118-1 (PMC6026144; doi:10.1038/s41598-018-28118-1)
Supplement: Supplementary file 1 — Supplementary information [file 41598_2018_28118_MOESM1_ESM.doc]

**Circulating miR-30a-5p as a prognostic biomarker of left ventricular dysfunction after acute myocardial infarction**

# Agata Maciejak^1^, Edyta Kostarska-Srokosz^2^, Wlodzimierz Gierlak^2^, Miroslaw Dluzniewski^3^, Marek Kuch^2^, Michal Marchel^4^, Grzegorz Opolski^4^, Marek Kiliszek^5^, Krzysztof Matlak^6^, ****Slawomir Dobrzycki^7^****, Anna Lukasik^1^, Agnieszka Segiet^8^, Grazyna Sygitowicz^9^, Dariusz Sitkiewicz^9^, Monika Gora^1*^, Beata Burzynska^1*^

^1^ Institute of Biochemistry and Biophysics, Polish Academy of Sciences, Warsaw, Poland

^2^ Department of Cardiology, Hypertension and Internal Medicine, Second Faculty of Medicine, Medical University of Warsaw, Warsaw, Poland

^3^ Cardiology Department, Midtown Medical Center, Mazovia Brodno Hospital

^4^ 1^st^ Chair and Department of Cardiology, Medical University of Warsaw, Warsaw, Poland

^5^ Department of Cardiology and Internal Diseases, Military Institute of Medicine, Warsaw, Poland

^6^ Department of Cardiac Surgery, Medical University of Bialystok, Bialystok, Poland

^7^ Department of Invasive Cardiology, Medical University of Bialystok, Bialystok, Poland

^8^ 1^st^ Faculty of Medicine, Medical University of Warsaw, Warsaw, Poland

^9^ Department of Clinical Chemistry and Laboratory Diagnostics, Medical University of Warsaw, Warsaw, Poland

* Corresponding authors:

email: atka@ibb.waw.pl; mgora@ibb.waw.pl

Institute of Biochemistry and Biophysics, Polish Academy of Sciences, Pawinskiego 5A, 02-106 Warsaw, Poland, Phone number: +4822 5921214, Fax number: +4822 6584636

Table S1. Spearman's rank correlation coefficients of miR-30a-5p level and NT-proBNP and LVEF values.

Table S2. Gene targets for miR-30a-5p expressed in heart and whole blood.

Table S3. GO enriched terms for heart expressed target genes.

Table S4. GO enriched terms for whole blood expressed target genes.
