# Supplementary material for: Circulating miR-30a-5p as a prognostic biomarker of left ventricular dysfunction after acute myocardial infarction
Source: Sci Rep. 2018 Jun 29;8:9883. doi: 10.1038/s41598-018-28118-1 (PMC6026144; doi:10.1038/s41598-018-28118-1)
Supplement: Supplementary file 2 — Table S1 [file 41598_2018_28118_MOESM2_ESM.doc]

Table S1. Spearman's rank correlation coefficients of miR-30a-5p level and NT-proBNP and LVEF values.

| miR-30a-5p | NT-proBNP | | LVEF | |
| --- | --- | --- | --- | --- |
|  | admission | six months after AMI | admission | six months after AMI |
|  | 0.020 (p = 0.854) | 0.169 (p = 0.128) | -0.159 (p = 0.153) | -0.226 (p = 0.040) |

Spearman's rank correlation coefficients were calculated for the miR-30a-5p level on admission and NT-proBNP and LVEF on admission and six months after AMI in the validation group (n = 85).
